# *In Situ* Ti6Al4V/TiB Composites Prepared by Hydrogen-Assisted Sintering of Blends Containing TiH_2_ and Ball-Milled Ti+TiB_2_ Powders

**DOI:** 10.3390/ma15031049

**Published:** 2022-01-29

**Authors:** Yuchao Song, Fucheng Qiu, Dmytro Savvakin, Xiaofeng Xu, Oleksandr Stasiuk, Orest Ivasishin, Tuo Cheng

**Affiliations:** 1 College of Materials Science and Engineering, Jilin University, Changchun 130025, China; songyc19@mails.jlu.edu.cn (Y.S.); qiufc21@mails.jlu.edu.cn (F.Q.); savva@imp.kiev.ua (D.S.); xuxiaofeng@jlu.edu.cn (X.X.); olek.stasiuk@gmail.com (O.S.); ivas@imp.kiev.ua (O.I.); 2 International Center of Future Science, Jilin University, Changchun 130025, China; 3 G.V. Kurdyumov Institute for Metal Physics, 03115 Kyiv, Ukraine

**Keywords:** titanium matrix composites, powder metallurgy, press-and-sinter, titanium boride

## Abstract

In the present study, 98.6–99.5% dense in situ reinforced Ti6Al4V/TiB composites were manufactured with a newly developed approach based on hydrogen-assisted blended elemental powder metallurgy (BEPM). The approach includes the activation milling of titanium powder produced with hydrogenation-dehydrogenation (HDH-Ti powder) with finer TiB_2_ additives, following blending with TiH_2_ and master alloy (MA) powders, and final press-and-sinter operations. Scanning electron microscope (SEM) observations prove the formation of microstructures with improved density and homogeneous distribution of TiB reinforcements in a sintered Ti6Al4V matrix. Hardness and compressive tests validated the high mechanical characteristics of produced composites. The effect of preliminary milling time over 2–6 h and the ratio of hydrogenated and non-hydrogenated titanium powders used (TiH_2_ vs. HDH Ti) on microstructure and mechanical properties were studied to further optimize the processing parameters. Test results indicate the above approach can be regarded as a promising route for the cost-effective manufacturing of Ti6Al4V/TiB composite with reduced porosity, tailored microstructure uniformity, acceptable impurity level and, hence, mechanical characteristics sufficient for practice applications.

## 1. Introduction

Metal matrix composites are the subject of numerous studies [1,2,3,4,5,6]. Particle-reinforced titanium matrix composites (PRTMCs) have been widely studied and have proved to be excellent materials for critical applications such as aerospace, defense and medical instruments due to isotropic homogeneity, improved modulus and high-temperature characteristics [1]. Among various reinforcements for such titanium-based composites, in situ formed TiB has been deemed as one of the reinforcements with the most potential owing to its high Young modulus, elevated specific strength, brilliant thermal stability and clean and strong metallurgical bonding interface with matrix [2,3]. Therefore, intensive efforts have been performed on manufacturing PRTMCs reinforced with in situ formed TiB. 

Compared with other manufacturing routes, Blended Elemental Powder Metallurgy (BEPM) provides a bottom cost level for PRTMC processing [4,5,6,7], especially if desirable microstructure and characteristics can be achieved with the simplest press-and-sinter option without using complicated and expensive technologies such as hot isostatic pressing [8,9] and spark plasma sintering (SPS) [10,11,12]. It has been demonstrated that using titanium hydride (TiH_2_) powder instead of titanium powder in the starting blends brings some advantages for the simple press-and-sinter production of PRTMCs. Using titanium hydride as a starting powder further promotes the cost-effectiveness of the BEPM manufacturing route, while TiH_2_ dehydrogenation with the emission of atomic hydrogen on vacuum heating can clean the powder surfaces, reducing the content of O, C and Cl in the final material [13]. Moreover, phase transformation of titanium hydride into dehydrogenated titanium induces the acceleration of diffusion and powder sintering activation. Unfortunately, for BEPM manufacturing of composites reinforced with in situ formed TiB particles, a positive influence of TiH_2_→Ti transformation on activated densification usually is not observed. The employment of raw TiB_2_ or B powders as the boron source in BEPM processing resulted in the formation of excessive residual porosity in the titanium-based matrix during Ti+TiB_2_→TiB or Ti+B→TiB reactions and inhomogeneous partially reacted reinforcements in sintered microstructures [14,15,16]. Another problem is that the achievement of fine TiB precipitations in sintered composites, because of coarse raw TiB_2_ particles or their agglomerations formed during powder preparation procedures, cannot be easily fractionized into dispersed ones. To improve the mechanical performance of composites, reduction of residual porosity and formation of fine boride reinforcements evenly distributed over the matrix is necessary. For the desirable modification of composite microstructure, application of hot isostatic pressing during sintering and post-sintering thermal mechanical processing should be performed. However, these extra procedures definitely bring unwanted increase in material cost. Previous work by current authors indicates that the hydrogen-assisted 2 stage press-and-sintering route can effectively transform the porous composites into nearly dense materials without hot pressing or hot deformation operations, but doubled powder preparation, compaction and sintering procedures induce risks for material contamination with atmospheric impurities and degraded ductile properties [17].

To achieve enhanced sintered densities (reduced porosities) of BEPM-produced titanium alloys, preliminary activation of raw powders or powder blends by milling can be successfully used [18,19,20]. The same treatment is a potential way to improve sintered microstructures of TiB reinforced composites. However, activation milling simultaneously increases the risk of powder contamination, which requires the proper selection of processing parameters to attain desirable characteristics of the final material. 

The main aim of the present study is to develop a cost-effective hydrogen-assisted BEPM press-and-sinter approach ensuring the formation of nearly dense composites on the base of a Ti6Al4V alloy matrix with uniformly distributed TiB particles and acceptable impurity contents. To achieve desirable characteristics during pressureless sintering, activation milling of powders was included in the processing. The impact of processing parameters on microstructure and mechanical properties of the produced composites is investigated to demonstrate the potential of the practice application of such a manufacturing approach.

## 2. Materials and Methods

**Figure 1:**
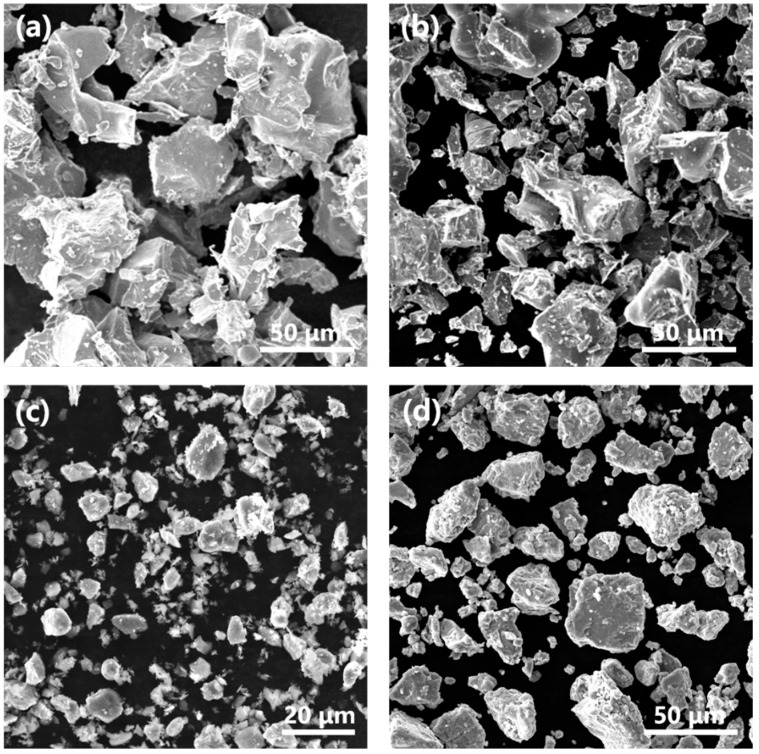
SEM-SE images of the starting powders: (**a**) HDH-Ti, (**b**) TiH_2_, (**c**) TiB_2_, (**d**) Al-V master alloy.

In the present research, BEPM production of composite on the base of a Ti6Al4V (wt%) alloy matrix reinforced with 5 vol.% of TiB phase was studied. Two kinds of powders, having different physical and mechanical characteristics, namely, TiH_2_ and HDH-Ti powders, were used together as the titanium base of powder blends. Titanium sponge (Baoti Huashen Titanium Industry Co., Ltd., Xi’an, China) was used as a starting material to produce both abovementioned powders. The sponge was hydrogenated up to a concentration of 3.5 wt% H (close to TiH_2_ composition) using heating to 600 °C and subsequent cooling to 300 °C under a hydrogen atmosphere. Then, the hydrogenated sponge was ball milled by a planetary milling device (QM-QX2, Nanda Instrument Plant, Nanjing, China) and sieved with 88 μm cell screen to obtain a TiH_2_ powder less than 88 µm in particle size. HDH-Ti powder (particle size 45–88 μm) was obtained by dehydrogenation of TiH_2_ powder (less than 88 µm) and 2nd sieving using a 45 µm screen to select desirable powder sizes. Apart from the above-noted two kinds of titanium source, relatively fine 60%Al-40%V master alloy (MA) (~10 μm) and TiB_2_ (~10 μm) powders (Futaiyuan metal materials Co., Ltd., Baoji, China) were used as starting materials. Figure 1 shows the actual morphology of the abovementioned raw powder particles based on SEM observations, while the impurity contents in raw materials are listed in Table 1.

Quite different characteristics of TiH_2_ and HDH Ti powders were profitably used in the manufacturing process of the Ti6Al4V-5%TiB composite to achieve a desirable, nearly dense and uniform microstructure. In various experiments (Table 2), an amount of TiB_2_ powder corresponding to 5 vol% TiB phase in final composite was preliminary ball milled for 2/4/6 h at 200 rpm together with various amounts of HDH Ti powder using a 5:1 ball to powder ratio. Despite the fact that ductile HDH Ti particles demonstrate a low tendency for size reduction with milling, it was assumed that the milling operation enables their severe plastic deformation and the covering of the surface of relatively large titanium particles with finer TiB_2_ ones. The close and tight bonding between TiB_2_ and HDH-Ti powders formed during milling should promote the activation of an in situ Ti+TiB_2_→TiB reaction at interfaces during the heating cycle and presumably suppress the formation of porosity. In contrast, brittle and low-strength TiH_2_ particles can be easily fragmented during milling, but the irregular morphology of hydride fragments (like that shown in Figure 1b) should not provide their close contact with TiB_2_ particles during milling and subsequent compaction. Moreover, intensive milling significantly increases the oxygen content in TiH_2_ powder [21]. For these reasons, preliminary activation milling of TiH_2_ powder was rejected. As was shown earlier, a positive contribution of titanium hydride to densification improvement can be achieved even without milling, owing to the additional fragmentation of TiH_2_ particles on compaction [22,23] and to the phase transformation of δ-TiH_2_ to β-Ti and α Ti on further vacuum sintering, which creates huge amounts of crystal lattice defects promoting the activation of diffusion as well as the homogenization and sintering of powder compacts controlled by diffusion [24]. Taking into consideration the above-described ideas, preliminary milled HDH-Ti+TiB_2_ powders were blended with corresponding amounts of TiH_2_ powder (Table 1) as well as Al-V MA powder to achieve total Ti6Al4V-5vol%TiB composition of each BEPM sample, while each sample (powder blend) was characterized with a definite TiH_2_-HDH-Ti ratio. To highlight the role of the individual TiH_2_ powder without the milling operation and, in contrast, the individually milled HDH Ti powder in the processing, blends 4 and 7, correspondingly, (Table 1) were prepared and tested. Powders were blended together for 6 h under argon atmosphere protection; powder blends were subsequently die-compacted at 600 MPa and sintered under vacuum at 1250 °C for 4 h to transform BEPM compacts into dehydrogenated bulk composites.

The effects of the processing parameters, including variation in milling time and different proportions of HDH-Ti used in the milling stage to TiH_2_ additions in the blending stage, on microstructural and mechanical characteristics of sintered composites were studied and corresponding samples were listed in Table 1.

Scanning electron microscopy under both secondary electron (SE) and backscattered electron (BSE) modes (SEM, JSM-IT500A, JEOL Ltd., Tokyo, Japan) was adopted for microstructure observation. Phase composition was identified using X-ray diffraction (XRD, Rigaku, Tokyo, Japan) (CuKa, λ = 0.15406 nm). Residual porosity was calculated as the ratio of actual density values measured following Archimedes’ method and theoretical density (4.44 g/cm^3^) of the composite. Hardness was determined using a HV 1000-IS hardness tester (Nuoen Ltd., Hangzhou, China) under the load of 1000 N holding for 10 s. Compressive tests were performed on cylindrical samples (6 mm in diameter and 9 mm in height) using an automatic universal testing machine at a constant crosshead speed of 1 µm/s. Oxygen contents in powders and in sintered composites were measured with a LECO ONH 836 analyzer (LECO Instruments Co., Ltd., St. Joseph City, AZ, USA). Each of the abovementioned tests was performed at least three times to validate the test results.

## 3. Results and Discussion

**Figure 2:**
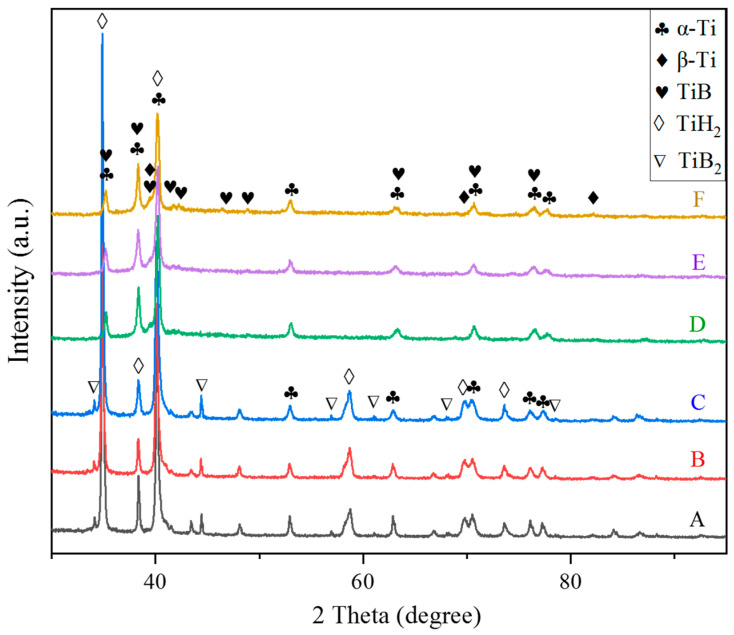
X-ray diffraction pattern of powder blends: (A) sample 1, (B) sample 2, (C) sample 3; and sintered composites: (D) sample 1, (E) sample 2, (F) sample 3.

**Figure 3:**
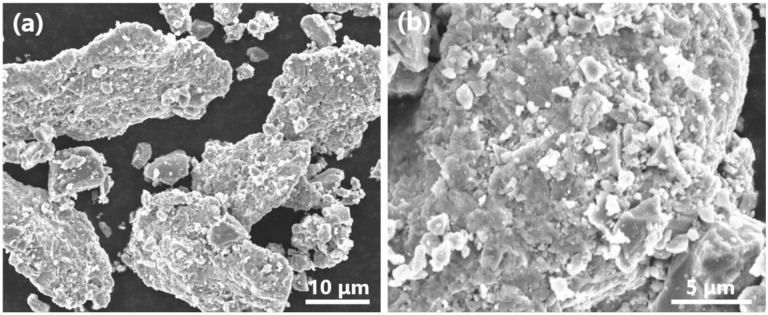
SEM-SE images of milled HDH-Ti+TiB_2_ powder blend at different magnifications. Blend milled for 4 h is presented as an example to show (**a**) typical morphology of particles and (**b**) TiB_2_ particles embedded into surface of Ti particle.

**Figure 4:**
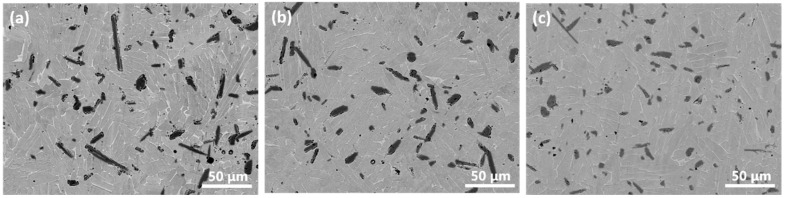
Microstructures (SEM-BSE) of composites produced with different preliminary milling times of HDH Ti+TiB_2_ powders: (**a**) 2 h, sample 1, (**b**) 4 h, sample 2, (**c**) 6 h, sample 3.

X-ray diffraction patterns of powder blends after varied preliminary milling time and corresponding sintered composites were shown in Figure 2. The presence of α-Ti (for HDH-Ti powder), TiH_2_ and TiB_2_ diffraction peaks without traces of boride intermediate phases for all powder blends indicates that no reaction between TiB_2_ and the matrix occurred even with the longest milling of 6 h. The absence of TiH_2_ and TiB_2_ peaks for all sintered composites (Figure 2D–F) reveals that TiH_2_ was totally dehydrogenated, while a α + β titanium matrix was formed. Apart from the TiB phases, no intermediate boride phases can be detected via diffraction patterns of sintered samples, proving the overall in situ transformation of raw TiB_2_ particles into TiB reinforcements via reaction with the titanium matrix (Ti+TiB_2_→TiB). Comparative observations on starting powders shown in Figure 1 and power blends after milling shown in Figure 3 demonstrated that finer and satellite-like TiB_2_ particles were tightly bonded on the surface of large HDH-Ti particles which were obviously deformed during milling. Deformed titanium particles should have an increased number of crystal lattice defects, which is useful for diffusion activation on further sintering. Moreover, preliminary milling not only is effective in the formation of close contacts between the surface of HDH Ti particles and TiB_2_ but also allows undesirable TiB_2_ agglomerations to be avoided and possibly results in the breaking of coarser TiB_2_ particles into finer ones with a more uniform redistribution of raw TiB_2_ phase over green powder blends. Changes of characteristics affected by different milling times are presented in Figure 4 and Table 3. Relatively large TiB needles distributed over the lamellar (α + β) matrix can be easily observed in sample 1, as shown in Figure 4a. However, the average length of TiB needles decreased when the milling time was increased (Table 3) from 28.7 μm for a 2 h milling (sample 1) to 21.4 μm for a 4 h milling (sample 2) and 18.9 μm for a 6 h milling (sample 3) without a noticeable difference in the cross-section size of TiB precipitations. On further sintering, diffusion redistribution of boron atoms in a titanium matrix resulted in corresponding more uniform TiB phase precipitations with a higher total number of TiB needles but which had shorter length. Furthermore, the residual porosity decreased with an extended milling time (Table 3, see samples 1–3 as an example). The composites were nearly fully consolidated in that a density of 99.5% of the theoretical value was achieved for sample 3. On the other hand, prolonged milling resulted in an increase in oxygen content which, in turn, affected hardness values (Table 3). Increased hardness of the composite was achieved with a longer milling time of HDH-Ti+TiB_2_ powders owing to the lower residual porosity, higher oxygen content and more uniform redistribution of TiB needles. Based on the fact that a similar porosity and TiB size were obtained for samples 2 and 3 (Table 3), the difference in their hardness (340–360 HV and 360–385 HV, correspondingly) can be explained mainly by the oxygen-induced strengthening effect.

So, an increase in preliminary milling time positively affects the uniformity of TiB reinforcement redistribution among the matrix and porosity reduction; both factors are useful for the improvement of strength and ductile characteristics. However, longer milling inevitably involves risks for the contamination of the powder blends with unwanted impurities even treated with argon protection, which is harmful for the ductile characteristics. To achieve impressive mechanical characteristics of manufactured composites, sintered materials require preservation of impurity content at the admissible level. Hence, a 4 h preliminary milling time is recommended as the optimized milling condition for the fabrication of Ti6Al4V/TiB composites with the milling-blending-sintering approach based on the overall analysis of the microstructure, hardness test results and impurity contents. 

**Figure 5:**
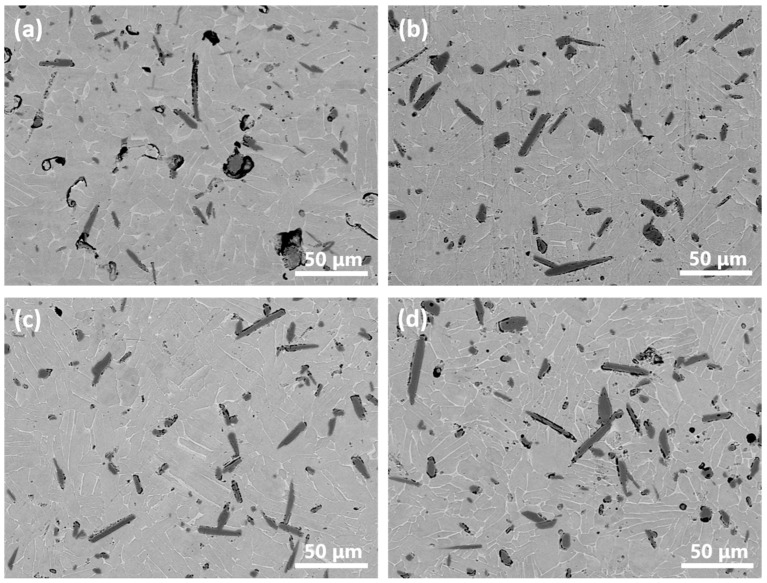
Microstructures of composites (SEM-BSE) produced with different HDH Ti-TiH_2_ proportions: (**a**) sample 4 (0:4), (**b**) sample 5 (1:3), (**c**) sample 6 (3:1), (**d**) sample 7 (4:0).

Reciprocal variations in HDH-Ti amount (used in the milling procedure) to TiH_2_ additions (used in the blending process) also significantly affected the characteristics of the manufactured Ti6Al4V-5%TiB composites. As shown in Figure 5a, equiaxed boride clusters surrounded with pores and coarse TiB needles were observed in the lamellar matrix for sample 4. Since neither the HDH-Ti powder nor milling process were involved for this sample, TiH_2_ alone was regarded as the matrix source. Apparently, a reaction between the dehydrogenated titanium matrix and coarsest TiB_2_ particles consumes large amounts of Ti atoms resulting in the formation of voids nearby; voids retard further reaction thus preserving the initial equiaxed morphology of not completely transformed boride particles, even though traces of residual TiB_2_ phase were not detected by X-ray analysis (Figure 2). Furthermore, mutual diffusion between the titanium matrix and Al-V MA particles during the homogenization of the powder blend can result in the formation of Kirkendall’s porosity, which is another possible contribution to the highest residual porosity observed (~4.3%, Table 3). In contrast, with the involvement of HDH-Ti in the milling procedure, the microstructure of samples 5–7 presents a lower porosity (1.1–1.4%, Table 3 and Figure 5b–d) with no presence of equiaxed boride clusters. So, it can be asserted that preliminary milling breaks the relatively larger TiB_2_ particles and agglomerations of TiB_2_ powders, creating close contact areas between boride and HDH-Ti particles which are sufficient for the complete Ti+TiB_2_→TiB reaction. The average length of reinforcing TiB needles is nearly similar for samples 5–7. It should also be mentioned that an increase in the amount of milled HDH-Ti powder with a corresponding decrease in the amount of TiH_2_ powder in the blend resulted in a higher oxygen content (Table 3). Sample 7, produced with a milled (HDH-Ti+TiB_2_) blend without titanium hydride additions, demonstrated the highest oxygen content (0.455%) as listed in Table 3 when compared with samples 2,4–6. This result once more confirmed the hydrogen cleaning effect achieved using TiH_2_ powder as well as the influence of the milling procedure on material contamination.

Clearly, the addition of TiH_2_ powder is necessary since dehydrogenation takes purification effect on all materials owing to the reaction of emitted hydrogen atoms and surface impurities. At the same time, to achieve the improved densification of powder blends, the advantages of TiH_2_ powder, such as the fragmentation of its particles by a compacting force and filling the voids in green compacts with small fragments [22,23] as well as activated diffusion due to the TiH_2_→Ti transformation [24], are less effective in the present case as compared to the activation milling of HDH-Ti+TiB_2_ powders (Table 3, samples 4–7). So, the separate use of either activation milling for HDH Ti+TiB_2_ powders (sample 7) or TiH_2_ powder (sample 4) resulted in compromised characteristics of sintered composites, such as an increased oxygen content and increased residual porosity, correspondingly. An acceptable combination of a highly dense sintered microstructure with evenly distributed TiB needles and admissible oxygen content was achieved when TiH_2_ and the activation milling of HDH-Ti+TiB_2_ powders were used together in various proportions of TiH_2_ and HDH Ti powders in the blends (Table 3, samples 2, 5, 6).

**Figure 6:**
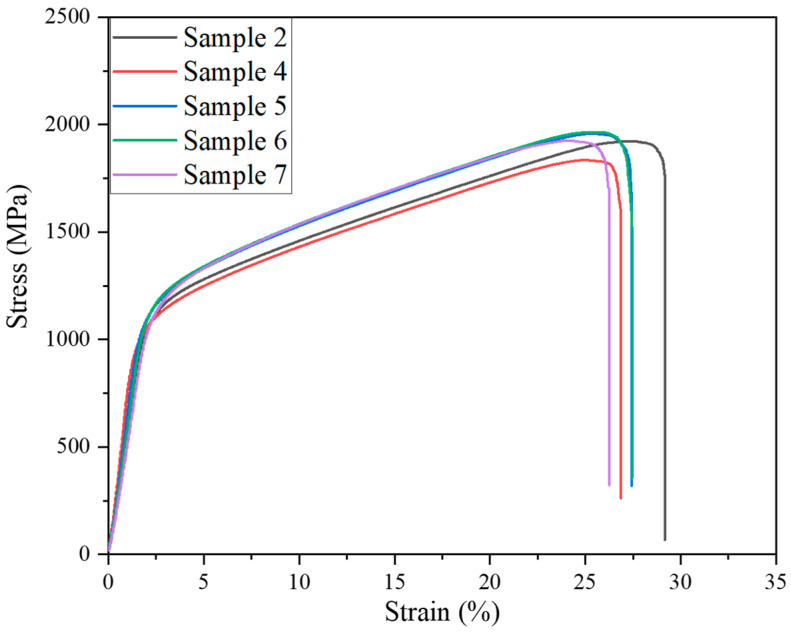
Engineering compressive strength–strain curves of composites produced with different HDH-Ti to TiH_2_ proportions.

Compressive testing results confirmed this conclusion. Figure 6 shows the typical engineering compressive stress–strain curves for composites produced using powder blends with different HDH-Ti to TiH_2_ proportions. The highest compressive strength levels (1965 ± 35 Mpa) were demonstrated by samples 5 and 6, while sample 2 presented the best compressive strain performance (29 ± 0.7%) at a slightly lower strength value (1923 ± 33 MPa). In contrast, the individual use of either HDH Ti or TiH_2_ powder (samples 4 and 7) resulted in noticeably reduced strength and strain characteristics. The deviations in compressive performance can be attributed to a mutual influence of several key factors. Higher proportions of milled HDH-Ti powder promote more contacting areas between severely deformed HDH-Ti and TiB_2_ powders, better pore healing on sintering and reduced residual porosity useful for the improvement of strength and strain characteristics. On the other hand, the decrease of TiH_2_ powder amount in the powder blend inhibited the cleaning effect induced by dehydrogenation during heating cycles. The oxygen pick-up during milling also contributes to hardness and strength improvement due to the oxygen strengthening mechanism, but negatively affects compressive strain performance as shown in Figure 6. Finally, important factors for mechanical characteristics are morphology, size of TiB phase and redistribution of reinforcing particles in the Ti6Al4V alloy matrix. Relatively fine TiB needles uniformly distributed in the sintered matrix (Figure 4b and Figure 5b,c) contributed to the improved strength and strain characteristics of samples 2, 5 and 6.

## 4. Conclusions

In the present study, Ti6Al4V-TiB composites exhibiting up to 99.5% density were produced using powder blends based on TiH_2_ and HDH Ti powders with relatively simple technological operations including activation milling, blending, cold compaction and vacuum sintering. The microstructure and characteristics of the composites were investigated depending on the variation of processing parameters, and the conclusions were drawn as follows.

- Preliminary activation milling of ductile HDH Ti powder and TiB_2_ particles over 2–6 h is more effective in the formation of nearly dense sintered composites than hydrogen-assisted sintering of non-milled TiH_2_+TiB_2_ powders, while application of titanium hydride provides the preservation of acceptable oxygen contents (0.31–0.35%) in sintered composites.
- An increase in the milling time of HDH-Ti+TiB_2_ powders within 2–6 h is useful for the reduction of residual porosity (2.3% down to 0.5%) and formation of finer TiB precipitations uniformly redistributed over the sintered matrix, but extended milling time negatively affect impurity content.
- Cooperative use of TiH_2_ and milled HDH-Ti particles as a titanium base of powder blends resulted in the improved mechanical characteristics of sintered composites. Variation of HDH-Ti-TiH_2_ ratio in powder blends affects residual porosity, microstructure uniformity and oxygen content of sintered composites allowing us to control their hardness and compressive characteristics.

## Figures and Tables

**Table 1:** Contents of oxygen, nitrogen and hydrogen in starting powders.

Powder	Oxygen (wt%)	Nitrogen (wt%)	Hydrogen (wt%)
TiH_2_	0.195	0.533	~3.5
HDH Ti	0.125	0.0134	0.00123
Master alloy	0.217	0.19	0.00213
TiB_2_	0.855	0.533	0.0089

**Table 2:** Variation of processing parameters used for manufacturing of Ti6Al4V-5 vol%TiB composites.

Powder Blend (Sample) No.	Duration of Pre-Milling (h)	HDH-Ti to TiH_2_ Ratio (Mass Fractions)	Sintering Regime
1	2	1:1	1250 °C 4 h
2	4	1:1	
3	6	1:1	
4	4	0:4	
5	4	1:3	
6	4	3:1	
7	4	4:0	

**Table 3:** Microstructural and hardness characteristics as well as oxygen content of sintered Ti6Al4V-5%TiB composites.

Blend No.	Average TiB Length (μm)	Hardness (HV)	Porosity (%)	Oxygen (wt%)
1	28.7	307.5 ± 7.5	2.3 ± 0.1	0.337
2	21.4	350 ± 10	0.6 ± 0.03	0.374
3	18.9	372.5 ± 12.5	0.5 ± 0.03	0.43
4	22.7	250 ± 10	4.3 ± 0.15	0.313
5	21.2	335 ± 10	1.4 ± 0.05	0.356
6	22.4	340 ± 10	1.1 ± 0.04	0.392
7	23.5	355 ± 5	1.2 ± 0.05	0.455

## Data Availability

Not applicable.

## References

[1] Falodun O.E., Obadele B.A., Oke S.R., Okoro A.M., Olubambi P.A. (2019). Titanium-based matrix composites reinforced with particulate, microstructure, and mechanical properties using spark plasma sintering technique: A review. Int. J. Adv. Manuf. Technol..

[2] Morsi K. (2019). Review: Titanium–titanium boride composites. J. Mater. Sci..

[3] Morsi K., Patel V.V. (2007). Processing and properties of titanium–titanium boride (TiBw) matrix composites—A review. J. Mater. Sci..

[4] Kumar M.S., Chandrasekar P., Chandramohan P., Mohanraj M. (2012). Characterisation of titanium–titanium boride composites processed by powder metallurgy techniques. Mater. Charact..

[5] Sadoun A.M., Mohammed M.M., Fathy A., El-Kady O.A. (2020). Effect of Al_2_O_3_ addition on hardness and wear behavior of Cu–Al_2_O_3_ electro-less coated Ag nanocomposite. J. Mater. Res. Technol..

[6] Sadoun A.M., Fathy A., Abu-Oqail A., Elmetwaly H.T., Wagih A. (2020). Structural, mechanical and tribological properties of Cu–ZrO_2_/GNPs hybrid nanocomposites. Ceram. Int..

[7] Fang Z.Z., Paramore J.D., Sun P., Chandran K.S.R., Zhang Y., Xia Y., Cao F., Koopman M., Free M. (2018). Powder metallurgy of titanium – past, present, and future. Int. Mater. Rev..

[8] Cai C., Song B., Qiu C., Li L., Xue P., Wei Q., Zhou J., Nan H., Chen H., Shi Y. (2017). Hot isostatic pressing of in-situ TiB/Ti-6Al-4V composites with novel reinforcement architecture, enhanced hardness and elevated tribological properties. J. Alloy. Compd..

[9] Cai C., He S., Li L., Teng Q., Song B., Yan C., Wei Q., Shi Y. (2019). In-situ TiB/Ti-6Al-4V composites with a tailored architecture produced by hot isostatic pressing: Microstructure evolution, enhanced tensile properties and strengthening mechanisms. Compos. Part B Eng..

[10] Sedehi S.M.R., Khosravi M., Yaghoubinezhad Y. (2021). Mechanical properties and microstructures of reduced graphene oxide reinforced titanium matrix composites produced by spark plasma sintering and simple shear extrusion. Ceram. Int..

[11] Singh N., Ummethala R., Karamched P.S., Sokkalingam R., Gopal V., Manivasagam G., Prashanth K.G. (2021). Spark plasma sintering of Ti6Al4V metal matrix composites: Microstructure, mechanical and corrosion properties. J. Alloy. Compd..

[12] Vasanthakumar K., Ghosh S., Koundinya N., Ramaprabhu S., Bakshi S.R. (2019). Synthesis and mechanical properties of TiCx and Ti(C,N) reinforced Titanium matrix in situ composites by reactive spark plasma sintering. Mater. Sci. Eng. A.

[13] Savvakin D.H., Humenyak M.M., Matviichuk M.V., Molyar O.H. (2012). Role of Hydrogen in the Process of Sintering of Titanium Powders. Mater. Sci..

[14] Ivasishin O.M., Markovsky P.E., Savvakin D.G., Stasiuk O.O., Rad M.N., Prikhodko S.V. (2019). Multi-layered structures of Ti-6Al-4V alloy and TiC and TiB composites on its base fabricated using blended elemental powder metallurgy. J. Mater. Process. Technol..

[15] Ivasishin O., Bagliuk G., Stasiuk O., Savvakin D.G. (2017). The Peculiarities of Structure Formation upon Sintering of TiH_2_+TiB_2_ Powder Blends. Phys. Chem. Solid State.

[16] Selvakumar M., Chandrasekar P., Ravisankar B., Balaraju J.N., Mohanraj M. (2015). Mechanical Properties of Titanium–Titanium Boride Composites through Nanoindentation and Ultrasonic Techniques—An Evaluation Perspective. Powder Metall. Met. Ceram..

[17] Song Y., Dong S., Stasiuk O., Savvakin D., Ivasishin O. (2020). Synthesis of Ti/TiB Composites via Hydrogen-Assisted Blended Elemental Powder Metallurgy. Front. Mater..

[18] Schumann E., Silvain J.-F., Bobet J.-L., Bardet M., Lu Y., Kotousov A., Lamirand-Majimel M. (2016). The effects of ball milling and the addition of blended elemental aluminium on the densification of TiH_2_ power. Mater. Chem. Phys..

[19] Fouzia H., Mamoun F., Naouel H., Linda A., Goussem M., Said M., Mohammed A.S., Alex M., Iost A., Weiß S. (2021). The effect of milling time on the microstructure and mechanical properties of Ti-6Al-4Fe alloys. Mater. Today Commun..

[20] Panek J., Karolus M. (2020). Structural studies on Ti–Cu alloy obtained by high-energy ball milling. Mater. Sci. Technol..

[21] Ivasishin O.M., Anokhin V.M., Demidik A.N., Savvakin D.G. (2000). Cost-Effective Blended Elemental Powder Metallurgy of Titanium Alloys for Transportation Application. Key Eng. Mater..

[22] Dong S., Ma G., Lei P., Cheng T., Savvakin D., Ivasishin O. (2021). Comparative study on the densification process of different titanium powders. Adv. Powder Technol..

[23] Dong S., Wang B., Song Y., Ma G., Xu H., Savvakin D., Ivasishin O. (2021). Comparative Study on Cold Compaction Behavior of TiH_2_ Powder and HDH-Ti Powder. Adv. Mater. Sci. Eng..

[24] Ivasishin O., Moxson V., Qian M., Froes F.H.S. (2015). 8-Low-cost titanium hydride powder metallurgy. Titanium Powder Metallurgy.

